# Effects of different surgical approaches on health-related quality of life in pediatric and adolescent patients with papillary thyroid carcinoma

**DOI:** 10.1007/s12672-024-00920-6

**Published:** 2024-03-02

**Authors:** Yanling Su, Feng Wang, Shunjin Chen, Xiyu Yao

**Affiliations:** 1 Developmental Behavioral Pediatrics Department, Fujian Children’s Hospital (Fujian Branch of Shanghai Children’s Medical Center), Fuzhou, Fujian China; 2 College of Clinical Medicine for Obstetrics & Gynecology and Pediatrics, Fujian Medical University, Fuzhou, Fujian China; 3 Department of Head and Neck Surgery, Clinical Oncology School of Fujian Medical University, Fujian Cancer Hospital, Fuzhou, Fujian China

## Abstract

**Objective:**

We aimed to compare the health-related quality of life (HRQOL) in pediatric and adolescent patients with papillary thyroid carcinoma who underwent bilateral or unilateral thyroidectomy.

**Methods:**

This prospective observational study recruited children and adolescents diagnosed with papillary thyroid carcinoma at low risk of recurrence. Participants were categorized into bilateral or unilateral thyroidectomy groups. They were asked to complete one questionnaire regarding basic information and three others regarding quality of life. HRQOL data from patients who underwent different procedures at various time points were compared.

**Results:**

Thirty patients underwent unilateral thyroidectomy and 54 bilateral thyroidectomy (median age = 14.27 years). HRQOL of the unilateral thyroidectomy group was higher than the bilateral group.

**Conclusion:**

For children and adolescents with papillary thyroid carcinoma at low risk of recurrence, bilateral thyroidectomy is associated with a lower HRQOL than unilateral thyroidectomy. Surgeons should consider tumor impact and HRQOL when selecting the most appropriate surgical method.

**Supplementary Information:**

The online version contains supplementary material available at 10.1007/s12672-024-00920-6.

## Introduction

The annual incidence of thyroid cancer in children and adolescents is modest, with one to three cases per one million children [1]. Thyroid cancer is the leading cause of endocrine malignancies in children and adolescents, and its incidence is increasing [2]. The prognosis for thyroid cancer in children and adolescents is favorable, especially for papillary carcinoma [3]. Although thyroid cancer in children and adolescents is more likely to be multifocal, locally invasive, involve the lymph nodes, show distant metastasis, and have a high risk of recurrence, the overall survival is higher, and the prognosis is better than that in adults [4].

With advances in treatment, 5-year survival rates for childhood and adolescent cancers have increased significantly during the past few decades, and the number of survivors has grown steadily [5]. However, the increased risks for various somatic and psychiatric disorders, anxiety, depression, sleep disorders, and long-term socioeconomic consequences (e.g., reduced educational attainment and unemployment) negatively affect the quality of life of childhood and adolescent cancer survivors [6]. Given the favorable prognosis of thyroid cancer in children and adolescents, it is important to pay equal attention to the health-related quality of life (HRQOL) in this group.

Surgery is the preferred treatment for thyroid cancer in children and adolescents. At present, bilateral thyroidectomy (BT) and unilateral thyroidectomy (UT) are the main surgical methods. For most patients with papillary thyroid carcinoma, BT is recommended, involving the bilateral glandular lobes, vertebral lobes, and isthmus [7]. However, the scope of surgical resection for low-risk papillary thyroid carcinoma in children and adolescents remains controversial. The disease-free survival rate of children with low-risk papillary thyroid carcinoma undergoing UT is no lower than that of children undergoing BT [8]. BT increases the risk of surgery-related complications, whereas UT increases the risk of residual cancer, both of which are associated with decreased quality of life for patients with papillary thyroid carcinoma [9].

An increasing number of studies are focusing on the quality of life of patients with thyroid cancer [9–11]. However, to the best of our knowledge, few studies have focused on the quality of life of pediatric and adolescent patients with papillary thyroid carcinoma. No other study has examined the relationship between surgical approaches and HRQOL in these patients. The aim of this study was to compare the effects of different surgical approaches on HRQOL in pediatric and adolescent patients with papillary thyroid carcinoma at low risk of recurrence.

## Materials and methods

### Participants

Pediatric and adolescent patients diagnosed with papillary thyroid carcinoma at low risk of recurrence at our institution from November 2017 to November 2022 were included. Low risk of recurrence is defined as a tumor confined to the thyroid with or without a few small central cervical lymph node metastases [7]. This prospective observational study was approved by the Ethics Committee of Fujian Provincial Cancer Hospital and conducted in accordance with the ethical standards of the Helsinki Declaration of 1975, as revised in 1983. All patients and their parents or guardians provided written informed consent.

All patients were subjected to rigorous physical and laboratory examinations, including preoperative evaluations of history and contraindications. The inclusion criteria were as follows: (1) aged under 18 years, (2) unilateral or bilateral thyroidectomy with unilateral or bilateral central lymph node dissection, (3) pathology-confirmed papillary thyroid carcinoma at low risk of recurrence, (4) patients and their parents/guardians agreed to participate in the study, and (5) patients had adequate reading and writing skills. The exclusion criteria were as follows: (1) moderate to high risk of recurrence, (2) pathological non-papillary thyroid carcinoma, (3) history of previous thyroid surgery, (4) cognitive-behavioral disorders, and (5) visual impairment.

### Research methodology

All enrolled patients were categorized into the BT or UT group based on the type of surgery they underwent. BT and bilateral central lymph node dissection were performed in the BT group, and UT with isthmus and unilateral central lymph node dissection were performed in the UT group. Basic information was collected, such as name, age, sex, type of surgery, parental marital status, only-child status, tumor size, and the number of central lymph node metastases; these factors were considered potential confounders. After surgery, participants were asked to complete questionnaires at 1, 3, 6, and 12 months postoperatively according to the inclusion and exclusion criteria. All questionnaires were completed via electronic mail, social media, or onsite. The first survey asked for basic information, and the other three were quality-of-life questionnaires: the Thyroid Cancer-Specific Quality of Life Questionnaire (THYCA-Qol), Pediatric Quality of Life Inventory (PedsQL), and the European Organization for Research and Treatment of Cancer Quality of Life Questionnaire (EORTC QLQ-C30) (version 3.0).

### Statistical analysis

The collected data were imported to SPSS (version 20.0, IBM, Armonk, NY, USA) for data processing. Measurement data are expressed as means ± standard deviations (x ± s). The t-test was used to compare normally distributed data, and the Wilcoxon rank-sum test (nonparametric test) was used for non-normally distributed data. Count data are expressed as frequencies and percentages and were analyzed using the χ^2^ test. *P*-values < 0.05 were considered statistically significant.

## Results

### Patient characteristics

A total of 96 patients were enrolled in this study. Twelve patients were lost during follow-up or withdrew from the study before completion; accordingly, 84 patients were included. The median age of the participants was 14.27 years. By comparing the basic information of the two groups (BT and UT), no statistically significant difference was observed between them in terms of sex, age, parental marital status, only-child status, tumor size, and number of central lymph node metastases (Table 1).

**Table 1:** Comparison of baseline information between unilateral and bilateral thyroidectomy groups (n = 84)

General	No. (%)		*P*
	UT (n = 30)	BT (n = 54)	
Age (years, x ± s)	14.07 ± 3.62	14.38 ± 3.54	0.28
Sex			
Boys	13 (43.3%)	15 (27.8%)	0.15
Girls	17 (56.7%)	39 (72.2%)	
Whether parents are divorced^a^			
Yes	5 (16.7%)	6 (11.1%)	0.70
No	25 (83.3%)	48 (88.9%)	
Only child			
Yes	16 (53.3%)	34 (63.0%)	0.39
No	14 (46.7%)	20 (37.0%)	
Tumor size (cm)	0.60 ± 0.21	0.54 ± 0.26	0.58
Number of central lymph node metastasis	0.37 ± 0.89	0.41 ± 1.65	0.79

^**a**^Theoretical frequencies < 5 but ≥ 1 and n ≥ 40 were tested with a continuity-corrected chi-square test. *BT* bilateral thyroidectomy, *UT* unilateral thyroidectomy, x ± s: mean ± standard deviation

### THYCA-Qol outcomes

**Fig. 1:**
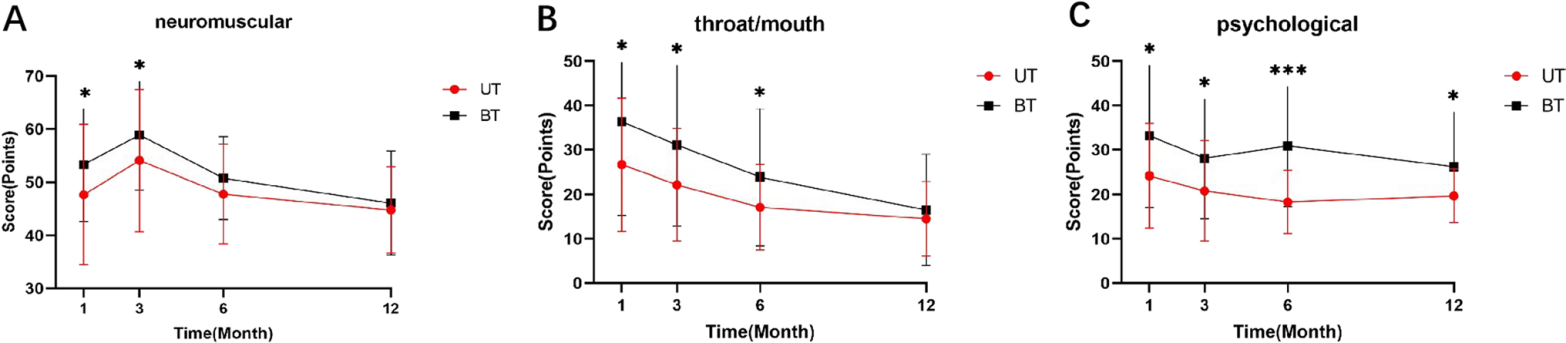
THYCA-Qol results in the UT and BT groups. **a** Score results for neuromuscular symptoms in both groups. **b** Score results for throat/mouth symptoms in both groups. **c** Score results for psychological symptoms in both groups. THYCA-Qol: Thyroid Cancer-Specific Quality of Life Questionnaire, *BT* bilateral thyroidectomy, *UT* unilateral thyroidectomy. *P < 0.05, **P < 0.01, ***P < 0.001

The THYCA-Qol results (Additional file 1: Online Resource 1) were compared between the two groups. The THYCA-Qol assessment consists of seven symptom domains (neuromuscular, voice, concentration, sympathetic, throat/mouth, psychological, and sensory) and five single items (scar, chilly, tingling, weight gain, headache, and decreased libido), with lower scores associated with better quality of life [12]. The entry for decreased libido was deleted because the participants were children and adolescents. In terms of neuromuscular symptoms (Fig. 1a), the UT group had significantly lower scores than the BT group at 1 (*P* = 0.02) and 3 months (*P* = 0.03) postoperatively, whereas there were no statistically significant differences at 6 and 12 months postoperatively. In terms of throat/mouth symptoms (Fig. 1b), the UT group had significantly lower scores than those of the BT group at 1 (*P* = 0.02), 3 (*P* = 0.01), and 6 months (*P* = 0.04) postoperatively, whereas there was no statistically significant difference at 12 months postoperatively. In terms of psychological symptoms (Fig. 1c), the UT group had significantly lower scores than those of the BT group at 1 (*P* = 0.01), 3 (*P* = 0.04), 6 (*P* < 0.00), and 12 months (*P* = 0.02) postoperatively. No statistically significant differences were observed between the scores of the two groups on each entry.

### PedsQL Outcomes

**Fig. 2:**
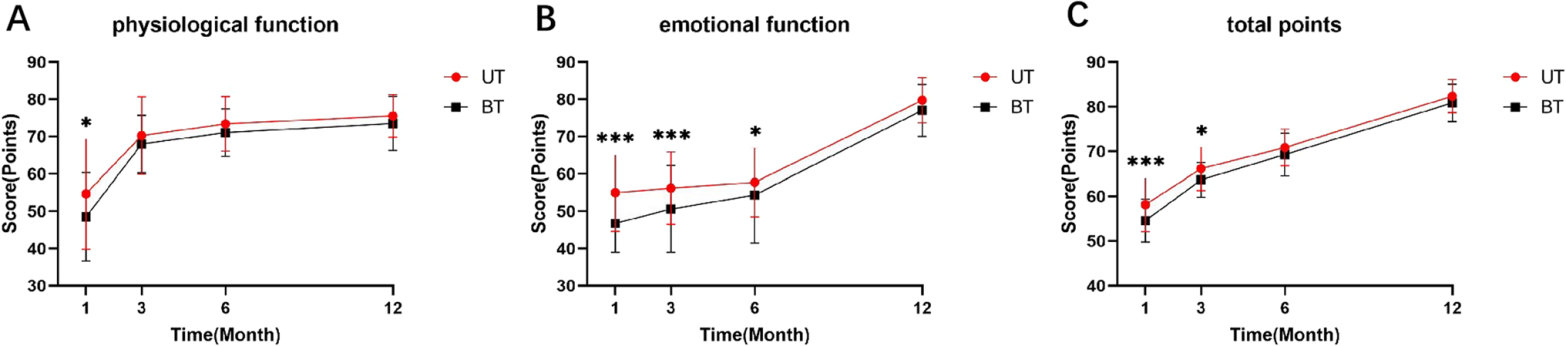
PedsQL results in the UT and BT groups. **a** Score results for physiological function in the two groups. **b** Score results for emotional function in the two groups. **c** Scale total score for the two groups. *PedsQL* Pediatric Quality of Life Inventory, *BT* bilateral thyroidectomy, *UT* unilateral thyroidectomy. *P < 0.05, **P < 0.01, ***P < 0.001

The results of the PedsQL (Online Resource 2) were compared between the two groups. The scale was categorized based on four dimensions: physiological function, emotional function, social function, and academic performance; the higher the score, the better the quality of survival [13]. Regarding physiological function (Fig. 2a), the UT group scored significantly higher than the BT group 1 month after surgery (*P* = 0.04), while there were no statistically significant differences in the scores at 3, 6, and 12 months after surgery. Regarding emotional function (Fig. 2b), the UT group had significantly higher scores than the BT group at 1 (*P* < 0.00), 3 (*P* < 0.00), and 6 months (*P* = 0.01) postoperatively, while the difference was not statistically significant at 12 months postoperatively. In terms of the total scale scores (Fig. 2c), the UT group had significantly higher scores than the BT group at 1 (*P* < 0.00) and 3 months (*P* = 0.01) postoperatively, but there were no statistically significant differences at 6 and 12 months postoperatively. No statistically significant differences were seen between the two groups regarding social function scores at any time point.

### EORTC QLQ-C30 Outcomes

**Fig. 3:**
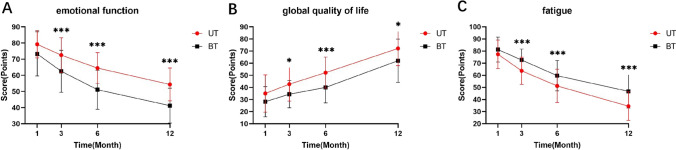
EORTC QLQ-C30 results in the UT and BT groups. **a** Score results for emotional function in both groups. **b** Score results for global quality of life in both groups. **c** Score results for fatigue in both groups. *EORTC QLQ-C30* European Organization for Research and Treatment of Cancer Quality of Life Questionnaire, *BT* bilateral thyroidectomy, *UT* unilateral thyroidectomy. *P < 0.05, **P < 0.01, ***P < 0.001

The results of the EORTC QLQ-C30 (Online Resource 3) were compared between the two groups of patients. The EORTC QLQ-C30 was used to measure the quality of life in patients with all cancers and consisted of five functional scales (physical, role, cognitive, emotional, and social), one global quality of life, and nine single-item scales (fatigue, nausea and vomiting, pain, dyspnea, sleep disturbance, appetite loss, constipation, diarrhea, and financial difficulties). Higher scores on the five domains of functioning and global quality of life are associated with a better functional status; additionally, in the nine individual scales, higher scores implied more severe symptoms [14]. In terms of emotional function (Fig. 3a), the UT group had significantly higher scores than the BT group at 3 (*P* < 0.00), 6 (*P* < 0.00), and 12 months (*P* < 0.00) postoperatively, whereas the difference was not statistically significant at the first postoperative month. Regarding the global quality of life (Fig. 3b), the UT group had significantly higher scores than the BT group at 3 (*P* = 0.01), 6 (*P* < 0.00), and 12 months (*P* = 0.01) postoperatively, while there was no statistically significant difference between groups at the first postoperative month. Regarding the single item of fatigue (Fig. 3c), the UT group had significantly lower scores than the BT group at 3 (*P* < 0.00), 6 (*P* < 0.00), and 12 months (*P* < 0.00) postoperatively, but there were no statistically significant differences at the first postoperative month. No statistically significant difference existed between the scores of the two groups for the remaining functional scales and single items.

## Discussion

This study aimed to compare the HRQOL of patients with papillary thyroid carcinoma at low risk of recurrence who underwent different thyroid surgery modalities. With the paradigm shift in modern medicine, where treatment emphasizes curing the patient’s disease and improving quality of life, there has been an increased focus on HRQOL, especially in children and adolescents, to assess health outcomes [15]. HRQOL is an important aspect of assessing health that cannot be detected by traditional physiologic and clinical examinations. The World Health Organization defines quality of life as the “perceptions of individuals of their place in life in the context of the culture and value system in which they live, as well as their goals, aspirations, standards, and concerns.” [16] HRQOL is a multidimensional construct with many subdimensions of subjective experience, including physical activity, mental health, social interaction, and school performance. In short, HRQOL reflects an individual’s self-assessment and perception of well-being, enjoyment, satisfaction with life, general health, and functioning [17].

Although the incidence of papillary thyroid carcinoma is low in children and adolescents, its prevalence has increased in recent years and has become the second most common cancer in adolescent girls aged 15–19 years[18]. Papillary thyroid carcinoma in children and adolescents has the highest 5-year survival rate at approximately 98%, compared with 84% for all childhood cancers (notably, the 84% survival rate for overall cancer survival was increased by the inclusion of childhood papillary thyroid carcinoma) [19]. Considering the high-survival rate for children and adolescents with papillary thyroid carcinoma, there is a higher quality of life expectation compared with that of patients with other cancers.

Lamia et al. found no differences in the quality of life of patients with papillary thyroid carcinoma compared with that of patients with other cancers, but the quality of life was lower compared with that of healthy children [20]. The quality of life of children and adolescents with papillary thyroid carcinoma needs to be studied; however, to date, few studies have focused on the quality of life of this population.

The treatment of thyroid cancer includes surgical treatment and thyroid ablation [21]. The mainstay of treatment for papillary thyroid carcinoma in children and adolescents is surgical intervention. Surgical methods include bilateral thyroidectomy and unilateral thyroidectomy [22]. Nevertheless, the choice of surgical approach remains controversial, with debate centering around the extent of thyroidectomy. In 2015, the American Thyroid Association released the first edition of its guidelines for managing pediatric thyroid cancer that recommended BT for most children [7]. Amarasinghe et al. reported that the incidence of residual cancer may increase by 30% without BT [23]. Bilateral thyroidectomy has a risk of recurrent laryngeal nerve paralysis [24]. All these factors reduce the quality of life for children and adolescents with thyroid cancer. However, the scope of surgical resection in children with a low risk of papillary thyroid carcinoma recurrence remains controversial. The consensus statement on pediatric benign and malignant thyroid surgery by the American Association of Clinical Endocrinology and the American Head and Neck Society Endocrine Surgery Section emphasizes taking into account the favorable prognosis of pediatric papillary thyroid carcinoma and the need to minimize the risk of complications and maintain quality of life in low-risk children, wherein less extensive surgery should be considered [25].

Therefore, this study aimed to compare the HRQOL of patients with papillary thyroid carcinoma who underwent different thyroid surgery modalities. Three different HRQOL questionnaires were used in this study to assess the quality of life of the patients in a holistic manner.

The results showed that patients who underwent BT had a worse quality of life regardless of the assessment questionnaire. These patients had more psychological burdens, resulting in poorer emotional and mood responses. Therefore, when the surgical approach does not benefit the tumor treatment significantly, clinicians should consider achieving better HRQOL for their patients, choosing the most appropriate surgical approach, and avoiding overtreatment.

Owing to the favorable prognosis of papillary thyroid carcinoma, patients may undergo a gradual process of acceptance of psychological and psychosocial stress reduction. This ideology is consistent with our results that the HRQOL of patients in all groups gradually improved with time after surgery. Patients with papillary thyroid carcinoma need time to reestablish their HRQOL to preoperative levels; however, for some elements, it may not be possible to achieve preoperative status within 1 year.

To the best of our knowledge, this is the first study to analyze the impact of different thyroid surgery modalities on the HRQOL of pediatric and adolescent patients with papillary thyroid carcinoma. The current study utilized three different HRQOL questionnaires to provide a more comprehensive HRQOL assessment from different dimensions and promote early detection of HRQOL-related symptoms. To this end, healthcare professionals can provide timely humanistic care and better healthcare services to patients.

This study has some limitations. First, the sample size of our study was small. Second, the follow-up period was short, and a longer follow-up period was needed. Finally, the questionnaire is a subjective evaluation.

## Conclusion

For children and adolescents with papillary thyroid carcinoma at low risk of recurrence, BT is associated with a lower HRQOL than UT. In addition to tumor impact, surgeons should consider the patient’s HRQOL when selecting the most appropriate surgical method.

### Supplementary Information

Additional file1 (ZIP 63 KB)

## Data Availability

The data underlying this article will be shared upon reasonable request to the corresponding author.
