# Supplementary material for: Effects of different surgical approaches on health-related quality of life in pediatric and adolescent patients with papillary thyroid carcinoma
Source: Discov Oncol. 2024 Mar 2;15:55. doi: 10.1007/s12672-024-00920-6 (PMC10909002; doi:10.1007/s12672-024-00920-6)
Supplement: Supplementary file 1 — Additional file1 (ZIP 63 KB) [file 12672_2024_920_MOESM1_ESM.zip › Supplementary/Online_Resource__1.docx]

**Effects of different surgical approaches on health-related quality of life in pediatric and adolescent patients with papillary thyroid carcinoma**

Journal: *Discover Oncology*

**Yanling Su, Feng Wang, Shunjin Chen, Xiyu Yao**

***Corresponding author:**

Feng Wang

Department of Head and Neck Surgery, Clinical Oncology School of Fujian Medical University, Fujian Cancer Hospital, Fuma Rd, No.420, Fuzhou, Fujian Province, 350014, China

Email: [562796005@qq.com](mailto:562796005@qq.com)

**Online Resource 1:** THYCA-Qol outcomes in the two patient groups

| 12 months | *P* | 0.31 | 0.85 | 0.54 | 0.90 | 0.76 | 0.02 | 0.76 | 0.62 | 0.74 | 0.73 | 0.69 | 0.70 |
| --- | --- | --- | --- | --- | --- | --- | --- | --- | --- | --- | --- | --- | --- |
|  | BT (x±s) | 46.1±9.8 | 3.7±11.1 | 26.2±9.5 | 8.7±9.6 | 16.5±12.5 | 26.2±12.5 | 12.1±10.9 | 35.8±23.2 | 12.3±16.2 | 9.9±15.3 | 8.6±14.7 | 13.0±17.6 |
|  | UT (x±s) | 44.8±8.1 | 2.8±7.7 | 24.4±9.5 | 8.3±9.5 | 14.5±8.4 | 19.7±6.0 | 11.1±10.1 | 33.3±21.5 | 11.1±16.0 | 11.1±16.0 | 10.0±15.5 | 11.1±16.0 |
| 6 months | *P* | 0.14 | 0.56 | 0.73 | 0.21 | 0.04 | 0.00 | 0.87 | 0.60 | 0.92 | 0.89 | 0.63 | 0.74 |
|  | BT (x±s) | 50.8±7.8 | 8.6±15.8 | 32.1±11.6 | 11.7±9.5 | 23.9±15.5 | 30.9±13.6 | 13.1±12.3 | 38.9±25.7 | 13.0±16.4 | 10.5±15.6 | 10.5±15.6 | 15.4±19.1 |
|  | UT (x±s) | 47.8±9.4 | 6.7±12.8 | 31.1±12.2 | 8.9±8.5 | 17.1±9.6 | 18.3±7.1 | 12.8±12.1 | 43.3±29.2 | 13.3±16.6 | 10.0±15.5 | 12.2±16.3 | 16.7±19.1 |
| 3 months | *P* | 0.03 | 0.72 | 0.94 | 0.24 | 0.01 | 0.04 | 0.41 | 0.85 | 0.70 | 0.94 | 0.91 | 0.60 |
|  | BT (x±s) | 58.9±10.3 | 14.5±18.3 | 36.7±13.9 | 15.1±10.9 | 31.1±18.2 | 28.1±13.6 | 13.0±12.8 | 42.6±21.9 | 14.8±16.7 | 8.0±14.4 | 9.3±15.1 | 18.5±23.0 |
|  | UT (x±s) | 54.1±13.4 | 13.3±17.7 | 36.1±11.6 | 12.2±9.7 | 22.2±12.7 | 20.8±11.3 | 15.6±13.8 | 42.2±19.5 | 13.3±16.6 | 7.8±14.3 | 8.9±15.0 | 21.1±23.9 |
| 1 month | *P* | 0.02 | 0.71 | 0.78 | 0.17 | 0.02 | 0.01 | 0.77 | 0.45 | 0.28 | 0.47 | 0.83 | 0.82 |
|  | BT (x±s) | 53.3±10.7 | 22.8±23.8 | 40.7±16.4 | 19.8±12.6 | 36.4±21.1 | 33.2±16.1 | 14.8±15.1 | 45.7±26.9 | 13.0±16.4 | 8.6±14.7 | 6.2±13.1 | 14.8±21.1 |
|  | UT (x±s) | 47.7±13.2 | 22.2±26.4 | 39.4±14.2 | 15.6±12.3 | 26.7±15.0 | 24.2±11.8 | 13.9±15.2 | 50.0±25.9 | 17.8±19.0 | 11.1±16.0 | 5.6±12.6 | 15.5±21.0 |
|  |  | neuromuscular | voice | concentration | sympathetic | throat/mouth | psychological | sensory | scar | chilly | tingling | weight gain | weight gain |
|  |  | THYCA scales | | | | | | | THYCA scales | | | | |

BT: bilateral thyroidectomy, UT: unilateral thyroidectomy, THYCA-Qol: Thyroid Cancer-Specific Quality of Life Questionnaire. X± s: mean± standard deviation.
