# Supplementary material for: Effects of different surgical approaches on health-related quality of life in pediatric and adolescent patients with papillary thyroid carcinoma
Source: Discov Oncol. 2024 Mar 2;15:55. doi: 10.1007/s12672-024-00920-6 (PMC10909002; doi:10.1007/s12672-024-00920-6)
Supplement: Supplementary file 1 — Additional file1 (ZIP 63 KB) [file 12672_2024_920_MOESM1_ESM.zip › Supplementary/Online_Resource_2.docx]

**Effects of different surgical approaches on health-related quality of life in pediatric and adolescent patients with papillary thyroid carcinoma**

Journal: *Discover Oncology*

**Yanling Su, Feng Wang, Shunjin Chen, Xiyu Yao**

***Corresponding author:**

Feng Wang

Department of Head and Neck Surgery, Clinical Oncology School of Fujian Medical University, Fujian Cancer Hospital, Fuma Rd, No.420, Fuzhou, Fujian Province, 350014, China

Email: [562796005@qq.com](mailto:562796005@qq.com)

**Online Resource 2:** PedsQL outcomes in the two patient groups

| 12 months | *P* | 0.17 | 0.06 | 0.47 | 0.70 | 0.12 |
| --- | --- | --- | --- | --- | --- | --- |
|  | BT  (x±s) | 73.5±7.3 | 77.1±7.0 | 89.4±5.8 | 88.1±7.7 | 80.9±4.2 |
|  | UT  (x±s) | 75.6±5.7 | 79.8±6.1 | 90.7±5.4 | 87.5±7.2 | 82.4±3.7 |
| 6 months | *P* | 0.13 | 0.01 | 0.61 | 0.64 | 0.12 |
|  | BT  (x±s) | 71.1±6.4 | 54.4±12.9 | 73.5±7.2 | 77.0±10.2 | 69.3±4.8 |
|  | UT  (x±s) | 73.4±7.3 | 57.8±9.3 | 75.0±7.5 | 75.8±7.1 | 70.9±4.1 |
| 3 months | *P* | 0.25 | 0.00 | 0.64 | 0.73 | 0.01 |
|  | BT  (x±s) | 68.0±7.7 | 50.6±11.6 | 67.9±7.0 | 68.3±10.9 | 63.7±3.9 |
|  | UT  (x±s) | 70.3±10.4 | 56.2±9.7 | 68.2±7.6 | 67.8±7.8 | 66.2±4.9 |
| 1 month | *P* | 0.04 | 0.00 | 0.68 | 0.34 | 0.00 |
|  | BT  (x±s) | 48.5±11.9 | 46.8±7.8 | 65.6±7.3 | 61.2±14.4 | 54.6±4.8 |
|  | UT  (x±s) | 54.7±14.9 | 55.0±10.4 | 66.3±6.4 | 58.7±11.6 | 58.2±6.1 |
|  |  | physiological function | emotional function | social function | academic performance | total points |

BT: bilateral thyroidectomy, UT: unilateral thyroidectomy, PedsQL: Pediatric Quality of Life Inventory. X± s: mean± standard deviation.
