# Supplementary material for: Effects of different surgical approaches on health-related quality of life in pediatric and adolescent patients with papillary thyroid carcinoma
Source: Discov Oncol. 2024 Mar 2;15:55. doi: 10.1007/s12672-024-00920-6 (PMC10909002; doi:10.1007/s12672-024-00920-6)
Supplement: Supplementary file 1 — Additional file1 (ZIP 63 KB) [file 12672_2024_920_MOESM1_ESM.zip › Supplementary/Online_Resource_3.docx]

**Effects of different surgical approaches on health-related quality of life in pediatric and adolescent patients with papillary thyroid carcinoma**

Journal: *Discover Oncology*

**Yanling Su, Feng Wang, Shunjin Chen, Xiyu Yao**

***Corresponding author:**

Feng Wang

Department of Head and Neck Surgery, Clinical Oncology School of Fujian Medical University, Fujian Cancer Hospital, Fuma Rd, No.420, Fuzhou, Fujian Province, 350014, China

Email: [562796005@qq.com](mailto:562796005@qq.com)

**Online Resource 3:** EORTC QLQ-C30 outcomes in the two patient groups

| 12 months | *P* | 0.34 | 0.38 | 0.00 | 0.14 | 0.09 | 0.01 | 0.00 | 0.91 | 0.57 | 1.00 | 0.45 | 0.46 | 0.90 | 0.93 | 0.55 |
| --- | --- | --- | --- | --- | --- | --- | --- | --- | --- | --- | --- | --- | --- | --- | --- | --- |
|  | BT  (x±s) | 3.6±3.8 | 4.6±8.2 | 41.2±10.8 | 8.0±9.0 | 7.1±9.5 | 62.1±17.9 | 46.8±13.9 | 1.5±4.9 | 1.5±4.9 | 0.0±0.0 | 2.5±8.8 | 0.6±4.5 | 3.7±14.0 | 2.5±8.8 | 19.8±26.3 |
|  | UT  (x±s) | 4.4±4.0 | 6.1±8.2 | 54.4±10.2 | 11.7±10.9 | 11.1±11.0 | 72.2±14.2 | 34.5±11.8 | 1.7±5.1 | 2.2±5.8 | 0.0±0.0 | 1.1±6.1 | 0.0±0.0 | 3.3±13.4 | 3.3±13.4 | 16.7±25.9 |
| 6 months | *P* | 0.43 | 0.82 | 0.00 | 0.45 | 0.53 | 0.00 | 0.00 | 0.79 | 0.85 | 1.00 | 0.41 | 0.69 | 0.56 | 0.26 | 0.95 |
|  | BT  (x±s) | 8.3±5.5 | 6.2±8.1 | 51.1±12.1 | 18.8±13.0 | 19.1±15.7 | 40.0±12.8 | 59.7±12.6 | 4.3±7.4 | 5.6±8.6 | 0.0±0.0 | 5.6±12.5 | 4.3±11.3 | 1.9±10.1 | 1.9±10.1 | 21.0±28.5 |
|  | UT  (x±s) | 9.3±6.2 | 6.1±9.3 | 64.5±9.5 | 16.7±14.5 | 16.7±14.5 | 52.2±12.9 | 51.2±13.9 | 3.9±7.2 | 5.0±7.8 | 0.0±0.0 | 3.3±10.2 | 3.3±10.2 | 2.2±8.4 | 3.3±10.2 | 20.0±27.1 |
| 3 months | *P* | 0.21 | 0.74 | 0.00 | 0.29 | 0.96 | 0.01 | 0.00 | 0.91 | 0.89 | 0.46 | 0.93 | 0.52 | 0.43 | 0.86 | 0.85 |
|  | BT  (x±s) | 18.1±7.3 | 12.1±10.5 | 62.5±13.0 | 35.4±13.1 | 32.1±12.9 | 34.4±11.3 | 72.7±9.1 | 8.3±9.6 | 32.7±12.9 | 0.6±4.5 | 13.0±17.6 | 11.7±18.5 | 2.5±10.9 | 2.3±10.9 | 22.2±28.2 |
|  | UT  (x±s) | 20.2±7.5 | 13.3±12.7 | 72.5±10.8 | 31.1±18.4 | 32.2±15.1 | 42.8±14.0 | 63.7±11.3 | 7.8±8.5 | 33.5±11.2 | 0.0±0.0 | 12.2±16.3 | 13.3±16.6 | 6.7±22.1 | 2.2±8.4 | 21.1±28.4 |
| 1 month | *P* | 0.33 | 0.62 | 0.07 | 0.73 | 0.32 | 0.11 | 0.11 | 0.61 | 0.55 | 0.90 | 0.15 | 0.42 | 0.36 | 0.90 | 0.85 |
|  | BT  (x±s) | 22.2±7.9 | 26.5±11.4 | 73.3±13.7 | 47.8±13.8 | 44.1±13.4 | 28.2±12.5 | 81.3±10.3 | 20.4±11.0 | 58.3±15.8 | 2.5±8.8 | 27.8±25.7 | 23.5±26.4 | 3.1±11.7 | 2.5±8.8 | 21.0±26.9 |
|  | UT  (x±s) | 24.4±9.2 | 28.3±13.2 | 79.2±8.4 | 49.4±20.8 | 47.8±18.9 | 35.0±15.4 | 77.4±11.8 | 18.9±12.2 | 60.6±16.1 | 2.2±8.4 | 36.7±28.2 | 27.8±26.4 | 5.6±15.4 | 2.2±8.4 | 20.0±27.1 |
|  |  | physical | role | cognitive | emotional | social | global quality of life | fatigue | nausea and vomiting | pain | dyspnea | insomnia | appetite loss | constipation | diarrhea | financial difficulties |
|  |  | functional  scales | | | | |  | single-item scales | | | | | | | | |

BT: bilateral thyroidectomy, UT: unilateral thyroidectomy, EORTC QLQ-C30: European Organization for Research and Treatment of Cancer Quality of Life Questionnaire. X± s: mean± standard deviation.
